# Real-Time Visual Feedback Device Improves Quality Of Chest Compressions: A Manikin Study

**DOI:** 10.30476/BEAT.2020.83080

**Published:** 2020-07

**Authors:** João B Augusto, Miguel B Santos, Daniel Faria, Paulo Alves, David Roque, José Morais, Victor Gil, Carlos Morais

**Affiliations:** 1 *Department of Cardiology, Hospital Professor Doutor Fernando Fonseca, Lisbon, Portugal*; 2 *Institute of Cardiovascular Science, University College London, London, United Kingdom*; 3 *Barts Heart Centre, St Bartholomew’s Hospital, London, United Kingdom*; 4 *Department of Cardiology, Hospital dos Lusíadas, Lisbon, Portugal *

**Keywords:** Resuscitation, Basic life support, Simulation, Training

## Abstract

**Objective:**

To evaluate the impact of a real-time visual feedback device on Chest comparison (CCs) rate and depth delivered by healthcare professionals.

**Methods:**

In a simulated scenario a sensor was placed on a manikin's chest and connected to a defibrillator which provided real-time visual feedback on the rate and depth of CCs. Thirty-two healthcare professionals performed sequentially 5 cycles of 30 CCs without (FeedOFF) and with (FeedON) feedback. CCs with a depth between 50 and 60mm and a rate between 100 and 120cpm were considered optimal.

**Results:**

Visual feedback resulted in a significant increase in the proportion of CCs with optimal depth (median 8.7 [interquartile range 0.7–55.5]% FeedOFF vs 63.3 [17.6–88.1]% FeedON, p=0.002) and optimal rate (median 51.3 [1.3 – 81.3]% FeedOFF vs 68.3 [45.3 – 86.1]% FeedON, *p*=0.018). Overall, CCs were too shallow and too fast in the FeedOFF cycle. There was also a significant increase in optimal CCs (optimal depth and rate) with the use of the feedback device (from median 0.7 [0 – 26.9]% FeedOFF to 31.9 [3.6-59.9]% FeedON, *p*=0.001). Participants’ factors such as age, sex, body mass index, job or time since last CPR training did not have a significant impact on CPR quality.

**Conclusions:**

In the absence of visual feedback, there is a tendency towards lower depth and higher rate of CCs. The use of feedback technology significantly improves the quality of CCs.

## Introduction

Cardiopulmonary resuscitation (CPR) has a fundamental role in patients presenting with cardiac arrest (CA).[1,2] Although survival rates remain poor, high quality CPR has a significant prognostic impact following CA [1-4]. The American Heart Association (AHA) and European Resuscitation Council (ERC) guidelines for resuscitation emphasize the importance of high quality CPR with prespecified targets in terms of depth and rate of chest compressions (CCs) [3,4]. Indeed, optimal CCs have been associated with increased cerebral perfusion and a higher chance of return of spontaneous circulation [3-5].

Despite these recommendations, trained healthcare professionals still fail to deliver CPR within the established protocols. Furthermore, CPR performance also rapidly declines overtime during resuscitation [3,4,6]. A considerable number of devices has been developed to provide feedback on chest compressions during CPR in an attempt to improve CPR quality in both training and clinical settings [7-11]. One type of feedback device consists of a sensor placed over the sternum and beneath the hands of the CPR provider. When pressed, such sensor can give information about the CCs’ rate and depth, and the rescuer can then adjust CPR according to the feedback given (e.g. slow down if CCs are being delivered too fast). In this regard, manikin studies are useful for evaluating new technologies in CPR, as they assess the subjects’ performance and minimize external confounders. Thus, by implementing a simulated scenario, one can identify specific factors that can be improved. The aim of our study was to evaluate the impact of a real-time visual feedback device on the quality of chest compressions in a simulated scenario, according to international recommendations.

## Material and Methods

We conducted a single-center observational study designed to assess the effects of a real-time visual feedback system on the quality of CPR during simulated resuscitation. This study was carried out at Hospital Prof. Doutor Fernando Fonseca, Lisbon, Portugal, between November and December 2015. Our study was approved by the institutional review board and ethics committee.

*Participants*

We recruited 36 healthcare professionals (nurses and physicians) from the Cardiology Department (both Cardiology ward and Cardiac Intensive Care Unit). All participants were certified by the American Heart Association (AHA) as Basic Life Support (BLS) or Advanced Life Support (ALS) providers in the 2 years prior to the study. Exclusion criteria included: (1) severe obesity (defined as a body mass index (BMI) >40Kg.m^-2^), (2) pregnancy, (3) any complaint / disorder concerning vision or upper limbs and (5) previous experience with a CPR feedback device. The study was approved by the Institutional Review Board, all participants provided informed consent and were enrolled on the same day the study took place. Data collected for each participant included: age, sex, BMI (using Du Bois formula), job (physician / nurse) and time since last CPR training (>12 months versus ≤12 months).

*Study Protocol*

Upon entering the study room, prior to testing, each participant had the opportunity to read the study protocol. Then, all participants received an explanation on how the CPR feedback device works and had some time to familiarize with it (2 minutes). Afterwards, each subject practiced CPR under supervision without feedback device, during 5 minutes or until they declared that they felt sufficiently trained. CPR providers stood next to the manikin, which was placed on a firm and even surface without any mattresses underneath (to obviate the confounding factor of mattress compressibility), and its height was adjusted at the participant’s knees height. Subsequently, participants were instructed to perform 5 sets of 30 chest compressions (CCs) on the manikin, each set intercalated by 2 breaths delivered by a second investigator, according to international guidelines. Chest compressions were performed sequentially without (FeedOFF) and with feedback information (FeedON) from the device. After performing the first cycle of CCs FeedOFF (the display of the device was covered during this cycle), subjects took a 2-min break and then performed a second cycle of CCs with FeedON. During the FeedON cycle, the feedback monitor was uncovered and placed at subject’s eye level. Participants were instructed to adjust the rate (target between 100-120 CC/min) and depth (target between 50-60mm) of CCs according to the real-time visual information provided by the feedback monitor. After completion of the scenario, participants were asked not to inform others about the set-up.

*Material*

**Fig. 1:**
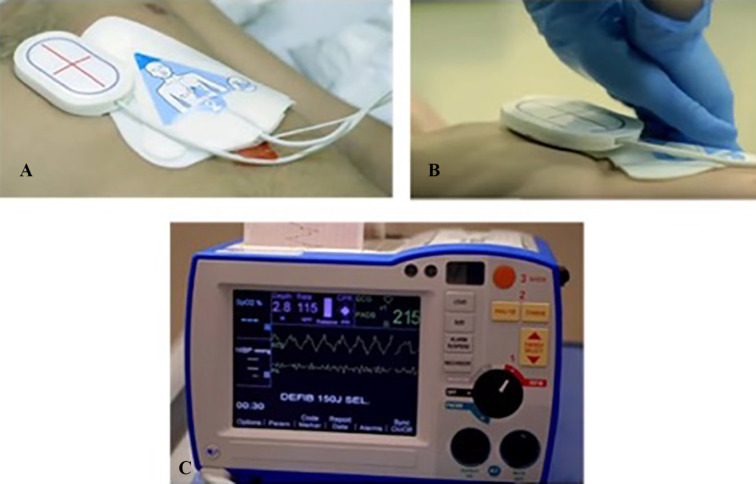
Set-up used in our study. One electrode was placed on the back of the manikin and the other electrode was placed over the manikin’s chest (A). The CPR sensor was positioned on the manikin’s sternum and beneath the hands of the rescuer (B). The electrodes were then connected to a defibrillator (C). *Image used and edited with permission of ZOLL Medical Corporation, Chelmsford, U.S*

**Fig. 2:**
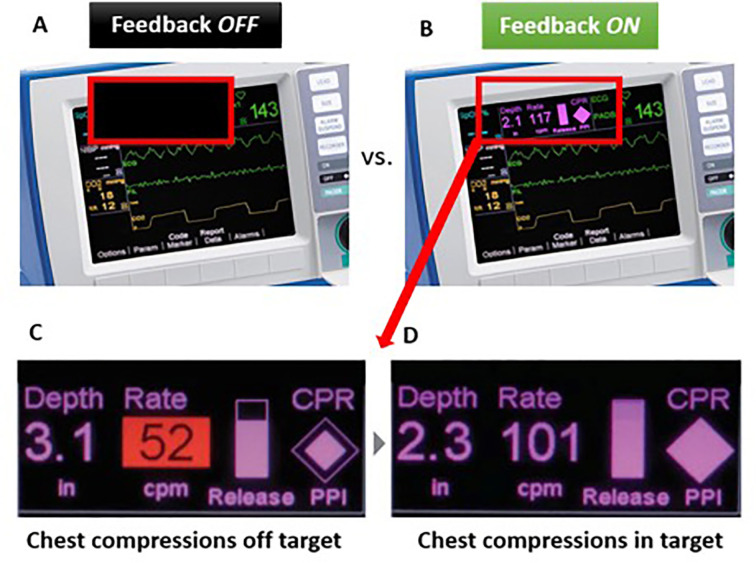
Defibrillator CPR Dashboard. During FeedOFF cycle (A), the CPR dashboard was covered and visual feedback information on CPR performance was not available. Throughout FeedON cycle (B), the CPR dashboard was uncovered and participants were instructed to adjust the compression rate (target 100 – 120cpm) and depth (target 50 – 60mm / 2.0 – 2.4 inches) according to the real-time visual feedback (C and D). *Image used and edited with permission of ZOLL Medical Corporation, Chelmsford, U.S*

**Fig. 3:**
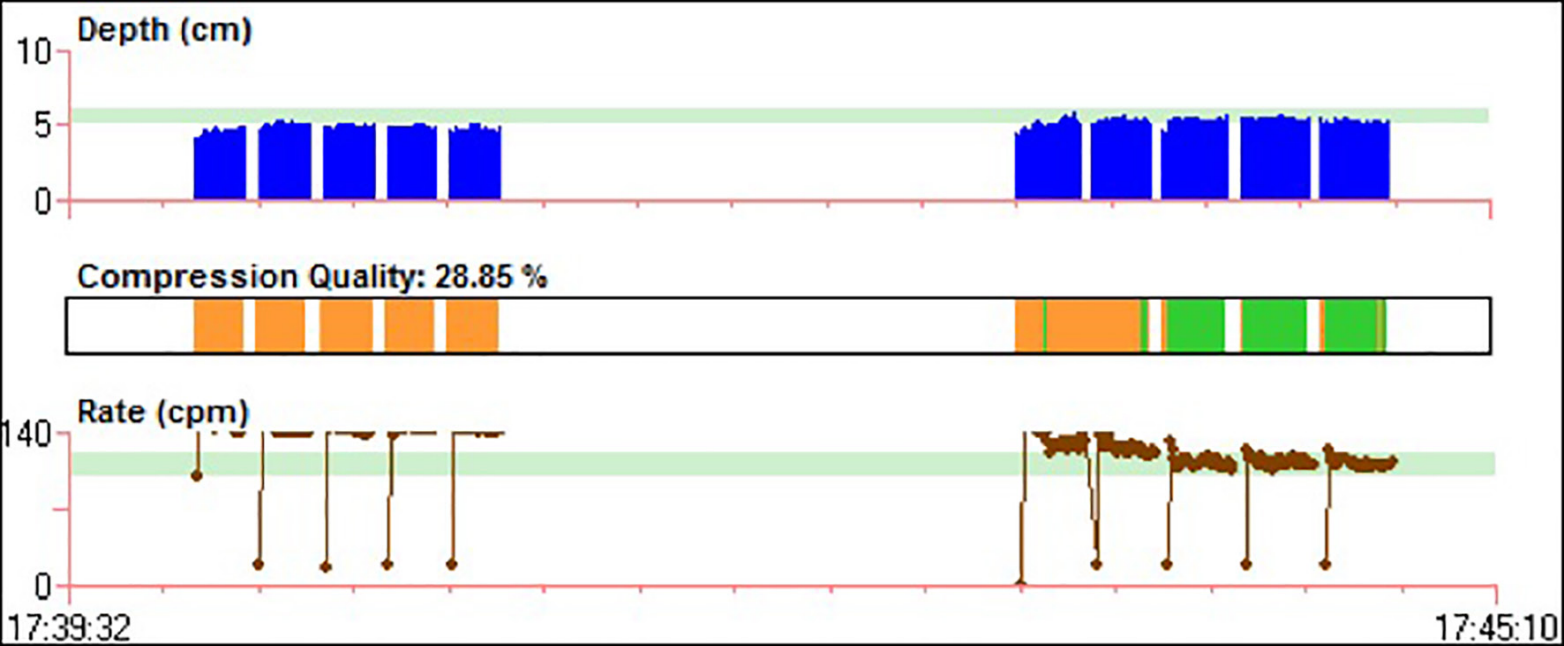
Analysis of CPR performance (participant #24). Interface of RescueNet Code Review^TM^ software: dark blue bars indicate depth (cm) of chest compressions (CCs), brown dots indicate rate of CCs (cpm), light blue horizontal bars specify CCs target zones (depth 50-60mm and rate 100-120cpm), orange bars designate CCs out of target (both depth and rate) and green bars indicate optimal CCs. During FeedOFF CPR cycle (left), the mean depth of CCs was 48.7mm (30.2% in target) and the mean rate of CCs was 142 cpm (0% in-target). During FeedON cycle (right), there was improvement in CPR performance – mean CCs depth was 52.8mm (56.4% in-target) and mean CCs rate was 117cpm (57.1% in target). Of note, in the initial phase of FeedON cycle, CCs were overall off-target. This could be partially explained by the initial adaptation and correction of CCs according to the information given by the feedback device. Overall compression quality (proportion of CCs with depth and rate in target) for both FeedOFF and FeedON cycles is reported – 28.85%.

We used the Adult Brad^TM^ manikin (Simulaids, Saugerties, U.S.), specifically designed and calibrated for CPR training. ZOLL OneStep™ Electrodes for adults were used for CPR quality measurements. These electrodes consist of a chest pad, a back pad and a CPR sensor which is placed over the manikin’s sternum and beneath the hands of the rescuer; the CPR provider then presses on the marked hand placement indicator that covers the sensor (Figures 1A and 1B). The electrodes are connected to a ZOLL R Series® defibrillator (Figure 1C) and the dashboard provides real-time visual feedback of the rate and depth of CCs during CPR (Figure 2). A ‘red flag’ appears when either rate or depth of CCs are off-target. Additionally, a visual diamond-shaped Perfusion Performance Index (PPI) provides a global assessment of both depth and rate of compressions; when the diamond is ‘full’ and no ‘red flags’ are present, chest compressions are optimal (within target). There is also a release velocity bar which indicates chest recoil after a compression; when the bar is full, the chest has been fully recoiled (however, release velocity was not evaluated in our study). A CPR idle timer starts if there are no CCs after a 3-second period. After completion of the protocol, data was transferred to a personal computer and RescueNet Code Review^TM^ Software (version 5.71, ZOLL Medical Corporation, Chelmsford, U.S.) was used for CPR performance analysis (Figure 3).

*CPR performance analysis*

We evaluated CPR performance for each cycle (FeedOFF and FeedON) according to the proportion of CCs with optimal rate (100-120 CC/min) or depth (50-60mm). Optimal CCs were defined as CCs with both rate and depth within target. We considered optimal resuscitation if at least 80% of CCs within a resuscitation cycle were optimal. Additionally, we calculated the change of CCs depth (Δdepth = mean depth during FeedON - mean depth during FeedOFF) and rate (Δrate = mean rate during FeedON - mean rate during FeedOFF) between FeedOFF and FeedON cycles. Then, we evaluated the impact of age, sex, BMI, job and time since last CPR training on Δdepth, Δrate and proportion of optimal CCs during Feed OFF and FeedON cycles. 

*Statistical analysis*

Discrete variables are presented as absolute frequencies with percentages and continuous variables as mean ± standard deviation if normally distributed, otherwise as median with interquartile range (IQR). Data were checked for normal distribution using Shapiro-Wilk test and visual Q-Q plots assessment. Measurements of depth and rate of CCs, as well as CPR performance (proportion of CCs with optimal rate or depth, and proportion of optimal CCs, both rate and depth) were compared between FeedOFF and FeedON cycles using Wilcoxon Signed Ranks Test. The effects of the feedback device on optimal resuscitation were assessed using McNemar’s test. The impact of subjective characteristics on Δdepth, Δrate and optimal CCs during Feed OFF and FeedON cycles was also evaluated; Pearson’s correlation coefficient was used for comparison between continuous data, and independent groups were compared using Students’ t-test for parametric data and Mann-Whitney U test for nonparametric data. All statistical analyses were performed using the SPSS for Windows version 22.0 (SPSS Inc., Chicago, IL, USA). Two-sided p-values <0.05 were considered statistically significant.

## Results

*Participants characteristics*

Out of 36 potential study participants, we excluded 2 due to upper limb’s musculoskeletal injury, one due to severe obesity and one due to pregnancy. A total of 32 participants were included in this study: median age was 34 (IQR 30.5 – 39.0) years, 68.8% male, 18.8% physicians, 81.2% nurses and 40.6% had their last CPR training >12 months before study participation. Median BMI was 22.0 (IQR 21.3 – 23.3) Kg.m^-2^. No dropouts occurred during study participation. All recorded data was valid for analysis by the RescueNet Code Review^TM^ Software. 

*CPR Performance*

**Fig. 4.**
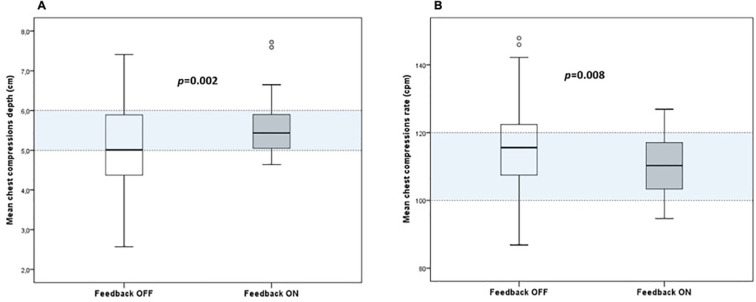
Chest compressions’ depth (A) and rate (B) during FeedOFF and FeedON cycles. The light blue area between two dashed lines represents the depth and rate target according to the 2015 AHA Guidelines

During FeedOFF cycle, mean depth of CCs was significantly lower than in FeedON cycle (5.1 ± 1.2cm vs 5.6 ± 0.8cm, *p*=0.002). The use of visual feedback resulted in a significant increase in the proportion of CCs with optimal depth (median 8.7 [IQR 0.7 – 55.5]% FeedOFF vs. 63.3 [17.6 – 88.1]% FeedON, *p*=0.002) (Figure 4A). Mean rate of CCs was significantly higher throughout the FeedOFF cycle than in the FeedON cycle (115.9 ± 14.0 vs. 110.2 ± 7.9cpm, *p*=0.008). The proportion of CCs with depth in target significantly increased with the use of visual feedback (51.3 [1.3-81.3]% FeedOFF vs. 68.3 [45.3 – 86.1]% FeedON, *p*=0.018) (Figure 4B). There was also a significant increment in optimal CCs with the use of the feedback device (from 0.7 [0 – 26.9]% FeedOFF to 31.9 [3.6 – 59.9]% FeedON, *p*=0.001). The results are summarized in Table 1. There was no significant improvement in terms of optimal resuscitations between FeedOFF and FeedON cycles (6.3 vs. 15.6%, respectively, p=0.250). The observed median percentage of too deep / too shallow compressions and too fast / too slow compressions are detailed in Table 2. 

We found no significant impact of participants’ age on Δdepth (Pearson’s correlation coefficient [PCC] r=-0.243, *p*=0.180), Δrate (PCC r=0.326, *p*=0.068) or proportion of optimal CCs FeedOFF (PCC r=-0.238, *p*=0.190) or FeedON (PCC r=-0.107, *p*=0.558). Similarly, BMI had no significant effect on Δdepth (PCC r=-0.172, *p*=0.347), Δrate (PCC r=-0.118, *p*=0.520) or optimal CCs FeedOFF (PCC r=-0.045, *p*=0.808) or FeedON (PCC r=-0.027, *p*=0.883). Proportion of optimal CCs during FeedOFF and FeedON cycles as well as Δdepth and Δrate were not significantly affected by subjects’ sex (*p*=0.325, *p*=0.826, *p*=0.732 and *p*=0.052, respectively), job (*p*=0.760, *p*=0.189, *p*=0.495 and *p*=0.381, respectively) or time since last CPR training (*p*=0.880, *p*=0.117, *p*=0.588 and *p*=0.188, respectively).

**Table 1:** Performance of chest compressions with and without visual feedback

** CPR** ^a^ ** performance**	**FeedOFF** ^b^	**FeedON** ^c^	***P*** **-value**
**Mean (SD) depth, cm**	5.1 (1.2)	5.6 (0.8)	0.002
**Median (IQR** ^d^ **) depth ** **in target** **, %** ^1^	8.7 (0.7 – 55.5)	63.3 (17.6 – 88.1)	0.002
**Mean ** **(SD** ^e^ **) ** **rate of compressions, cpm**	115.9 (14.0)	110.2 (7.9)	0.008
**Median (IQR) rate of compressions ** **in target** **, %** ^2^	51.3 (1.3 – 81.3)	68.3 (45.3 – 86.1)	0.018
**Median ** **(IQR)** ** optimal chest compressions, %** ^3^	0.7 (0 – 26.9)	31.9 (3.6 – 59.9)	0.001

^a^CPR: Cardiopulmonary Resuscitation; ^b^FeedOFF: Chest Compressions without visual feedback; ^c^FeedON: Chest compressions with visual feedback; ^d^IQR: Interquartile range; ^e^SD: Sandard deviation. (^1^Compression depth between 50 and 60mm; ^2^Compression rate between 100 and 120 compressions per minute; ^3^Compression depth between 50 and 60mm and compression rate between 100 and 120 compressions per minute)

**Table 2:** Proportion of chest compressions off target without (FeedOFF) and with (FeedON) visual feedback

** Off target chest compressions**	**FeedOFF** ^a^	**FeedON** ^b^	***P*** **-Value**
**Median (IQR** ^c^ **) chest compressions >60mm depth, %**	0 (0 – 60.2)	0 (0 – 30.5)	0.570
**Median (IQR) chest compressions <50mm depth, %**	26.8 (1.3 – 99.3)	7.7 (0.6 – 43.8)	<0.001
**Median (IQR) chest compressions >120cpm, %**	39.5 (4.2 – 90.9)	13.8 (1.6 – 47.2)	0.043
**Median (IQR) chest compressions <100cpm, %**	3.3 (2.7 – 5.6)	3.6 (3.3 – 6.1)	0.258

^a^FeedOFF: Chest compressions without visual feedback; ^b^FeedON: Chest compressions with visual feedback; ^c^IQR: interquartile range.

## Discussion

Our simulated CPR scenario showed that a visual feedback device significantly improves chest compression quality provided by healthcare professionals. The proportion of optimal compressions significantly increased, and these results are in line with most previous findings [7-11]. Zapletal *et al*. conducted a randomized prospective study comparing three CPR feedback devices and standard BLS and found no significant differences in effective compression rates in 240 study participants [12]. However, the study population consisted mostly of recently trained BLS providers (all groups had undergone several hours of CPR training without feedback), suggesting that the effect of these devices may be possible in a group of rescuers with insufficient or outdated training. In contrast, we found no differences in performance between healthcare professionals who were recently trained in BLS/ALS and those with outdated training. This finding leaves us to conclude that there is room for improvement, even in experienced or recently trained CPR providers. 

A worldwide effort is being made to standardize the quality of CPR. The European Resuscitation Council and the American Heart Association have identified the fundamental aspects of high-quality CPR: (1) minimize chest compression interruptions; (2) adequate compression rate and depth; (3) avoid leaning between compressions and (4) avoid excessive ventilation [3,4]. In our study, chest compressions during the simulated scenario were overall too shallow and too fast without the aid of the feedback device. These findings reinforce the need for CPR quality improvement. Healthcare providers typically overestimate the proportion of optimal CPR delivered [13]. One study conducted by Chang and colleagues demonstrated that the use of real-time feedback improves accuracy perception of CPR depth in CPR providers when compared with teamleaders [13]. A systematic review by Yeung and colleagues concluded that there was satisfying evidence supporting the use of real-time feedback devices during training to provide skill acquisition [14]. Manikin and human studies brought evidence on the use of real-time feedback devices and enabled a wide range of healthcare professionals to follow resuscitation guidelines more closely [15]. A panoply of methods have been proposed and developed to support healthcare professionals training, including audio-feedback systems,[16] video-recording systems, [17] automated external defibrillator interaction systems, [18] mobile applications, [19] metronomes [20] and motion sensing devices [21].

A visual feedback system was able to significantly improve the quality of chest compressions provided by healthcare professionals in a manikin simulated cardiac arrest scenario. The proportion of chest compressions with optimal depth and rate was significantly increased, even in recently trained CPR providers. The use of feedback systems in clinical practice can provide crucial information and help monitoring the performance of CPR providers. Further clinical trials are needed to evaluate the impact of such strategies in clinical practice and its outcomes in morbidity and mortality.

*Limitations*

Although study population was relatively small and there was no control group, the positive results were noteworthy. The use of a visual feedback device implies a constant visual focus with the equipment, which may be difficult in a real world scenario. Chest release parameters were not evaluated. Finally, a manikin trial does not mimic a real life CPR inasmuch there are important environment factors that cannot be reproduced in this kind of study: (1) stress levels are lower than in clinical practice; (2) CPR was performed only for a short period and, therefore, the fatigue factor was not reproduced; (3) healthcare providers only had to focus on chest compressions and there are significant distractors during a real life scenario (e.g. communication with the CPR team, defibrillation). 

In conclusion, a visual feedback system was able to significantly improve the quality of chest compressions provided by healthcare professionals in a manikin simulated cardiac arrest scenario. The proportion of chest compressions with optimal depth and rate was significantly increased, even in recently trained CPR providers. The use of feedback systems in clinical practice can provide crucial information and help monitoring the performance of CPR providers. Further clinical trials are needed to evaluate the impact of such strategies in clinical practice and its outcomes in morbidity and mortality.

## Funding:

The authors received no financial support for the research, authorship, and/or publication of this article.

## Conflicts of interest:

The authors have no conflicts of interest to declare.
